# Exercise capacity and handgrip strength following dumbbell-assisted diaphragmatic breathing training in patients with bronchiolitis obliterans syndrome after allogeneic hematopoietic stem cell transplantation: a prospective single-arm study

**DOI:** 10.3389/fmed.2026.1948656

**Published:** 2026-10-13

**Authors:** Xinyu Yuan, Wei Ge, Xiaojie Zhu, Yunxia Chen, Junhong Jiang, Huijun Zhang

**Affiliations:** 1 Department of Nursing, Jinzhou Medical University, Jinzhou, Liaoning, China; 2 Department of Respiratory and Critical Care Medicine, The Fourth Affiliated Hospital of Soochow University, Suzhou, Jiangsu, China

**Keywords:** 6MWT, allo-HSCT, BOS, dumbbell-assisted diaphragmatic breathing training, pulmonary rehabilitation

## Abstract

**Background:**

Bronchiolitis obliterans syndrome (BOS) is a severe complication after allogeneic hematopoietic stem cell transplantation (allo-HSCT) and is associated with impaired exercise capacity. Feasible rehabilitation strategies for this population remain limited.

**Objectives:**

To evaluate changes in functional exercise capacity and handgrip strength following a 12-week dumbbell-assisted diaphragmatic breathing training program in patients with BOS after allo-HSCT and to explore associations of these changes with selected baseline characteristics.

**Methods:**

This prospective, single-center, single-arm pre–post interventional study included 31 patients with BOS after allo-HSCT. Participants completed a 12-week dumbbell-assisted diaphragmatic breathing training program. Six-minute walk distance (6MWD) and bilateral handgrip strength were assessed at baseline and week 12. Within-participant changes were evaluated using paired tests according to the distribution of change scores. Associations with selected baseline characteristics were examined in exploratory analyses.

**Results:**

Following the 12-week intervention, mean 6MWD increased from 406.3 m at baseline to 425.9 m at week 12, corresponding to a mean within-participant increase of 19.6 m (95% CI: 9.3–29.9 m; *p* < 0.001). Mean bilateral handgrip strength increased from 23.2 kg to 25.0 kg, corresponding to a mean increase of 1.8 kg (95% CI: 1.3–2.3 kg; *p* < 0.001). The change in 6MWD did not differ significantly between women and men (*p* = 0.282), whereas the increase in handgrip strength was greater in women than in men (*p* = 0.021). Baseline heart-rate response during the 6MWT and supplemental oxygen requirement were not clearly associated with the magnitude of functional change. No serious training-related adverse events were observed.

**Conclusions:**

Twelve weeks of dumbbell-assisted diaphragmatic breathing training was associated with improvements in 6MWD and handgrip strength in patients with BOS after allo-HSCT. However, the single-arm design precludes causal attribution of these improvements to the intervention, and the exploratory subgroup findings should be interpreted cautiously. Larger controlled studies are needed to establish efficacy and characterize potential differences in response.

## Introduction

Allogeneic hematopoietic stem cell transplantation (allo-HSCT) is a cornerstone treatment for various hematological diseases. With continuous advances in transplantation techniques, supportive care, and immunosuppressive regimens, the long-term survival of patients after HSCT has been significantly improved (1–3). Currently, the prevention and management of chronic post-transplant complications have become a major clinical focus. Bronchiolitis obliterans syndrome (BOS) is a severe and devastating pulmonary complication following allo-HSCT. Its core pathological features include small airway epithelial injury, inflammatory cell infiltration and progressive fibrosis, which eventually lead to irreversible narrowing of small airways and fixed airflow obstruction, severely impairing pulmonary function and exercise capacity in patients (4, 5).

Epidemiological data show that the 5-year cumulative incidence of BOS after allo-HSCT is 3%–6.5% (6, 7). In patients complicated with chronic graft-versus-host disease (cGVHD), the incidence rises sharply to 14%–32%, making BOS one of the leading causes of late mortality after transplantation (5, 8). Patients with BOS mainly present with persistent cough, wheezing, progressive dyspnea, markedly reduced exercise tolerance, and recurrent lower respiratory tract infections, accompanied by respiratory and peripheral muscle atrophy, increased fatigue, and severely impaired quality of life (9).

Currently, the clinical management of BOS mainly relies on immunosuppressants and bronchodilators. Although these treatments can control airway inflammation and relieve dyspnea to a certain extent, they have very limited effects on improving impaired exercise tolerance, muscle strength, and respiratory function. Moreover, long-term immunosuppressive therapy is associated with risks such as infection and treatment-related organ toxicity and does not directly address the functional impairments that contribute to patients' long-term rehabilitation needs (10, 11). Therefore, safe and feasible rehabilitation strategies that address functional impairment beyond pharmacological management warrant further investigation.

Pulmonary rehabilitation is an evidence-based comprehensive intervention for chronic respiratory diseases, including exercise training, respiratory muscle training, health education, nutritional support, and psychological intervention (12–14). Pulmonary rehabilitation can improve exercise tolerance, symptoms, physical function, and quality of life in patients with chronic respiratory diseases, serving as a core intervention beyond pharmacological treatment (13, 14). As a key component of pulmonary rehabilitation, respiratory muscle training can enhance the strength and endurance of respiratory muscles (such as the diaphragm and intercostal muscles), reduce the work of breathing, relieve dyspnea, and thereby improve exercise tolerance in patients (15, 16).

Respiratory rehabilitation approaches may include breathing techniques such as pursed-lip and diaphragmatic breathing, respiratory-muscle training, and broader exercise-based pulmonary rehabilitation programs. Evidence from studies in chronic respiratory diseases suggests that respiratory-muscle training and exercise-based pulmonary rehabilitation can improve selected functional and respiratory outcomes, although the magnitude and nature of benefit vary according to the underlying disease, intervention, and training protocol (17, 18).

The intervention evaluated in the present study was dumbbell-assisted diaphragmatic breathing training (DADBT), in which diaphragmatic breathing was performed against resistance using an adjustable-resistance breathing trainer. Diaphragmatic breathing and respiratory-muscle training have been investigated as components of respiratory rehabilitation in different clinical populations (19–21). Mechanical respiratory-training devices can provide resistive loading and permit adjustment of the training load during respiratory-muscle training (22). The portability of device-assisted respiratory training may facilitate repeated practice outside specialized rehabilitation facilities, which could be particularly relevant for patients requiring prolonged rehabilitation or facing barriers to regular participation in center-based programs.

Home-based pulmonary rehabilitation has been investigated as an alternative mode of rehabilitation delivery for patients with chronic respiratory diseases, particularly when access to center-based programs is limited (23, 24). Previous studies of respiratory training after HSCT have generally involved small samples and have primarily evaluated conventional respiratory-muscle training approaches (25). Thus, evidence regarding structured respiratory rehabilitation in patients with BOS after allo-HSCT remains limited, and the potential role of this device-assisted diaphragmatic breathing program in improving functional outcomes in this population has not been specifically evaluated.

A recent retrospective study from our center evaluated nutritional support combined with structured respiratory training in a broader population with obliterative bronchiolitis and reported improvements in pulmonary and functional outcomes (26). However, that study differed from the present investigation in both population and intervention and did not specifically evaluate this portable device-assisted diaphragmatic breathing program in patients with BOS after allo-HSCT. Accordingly, the present prospective single-arm study evaluated changes in functional exercise capacity and handgrip strength following a 12-week dumbbell-assisted diaphragmatic breathing training program in patients with BOS after allo-HSCT. We also performed exploratory analyses to examine whether changes in these outcomes were associated with selected baseline demographic and clinical characteristics.

## Materials and methods

### Study design

This prospective, single-center, single-arm pre–post interventional study was conducted in the Department of Respiratory and Critical Care Medicine, The Fourth Affiliated Hospital of Soochow University, between August 2025 and February 2026. The study protocol was approved by the Ethics Committee of Jinzhou Medical University (approval No. JMU/EC/2025-03/182), and written informed consent was obtained in accordance with the approved protocol.

### Participants

The inclusion criteria were: (1) age 12–70 years; (2) BOS was diagnosed according to the 2014 National Institutes of Health (NIH) chronic graft-versus-host disease consensus criteria (27), including new-onset airflow obstruction defined by FEV_1_/VC <0.70 or below the fifth percentile of predicted, FEV_1_ <75% of predicted with a decline of ≥10% within 2 years, absence of active respiratory tract infection, and supporting evidence of air trapping or small-airway disease on expiratory high-resolution computed tomography or pulmonary-function testing; (3) clinically stable condition permitting participation in rehabilitation training and outcome assessments; and (4) ability to understand and follow study instructions.

The exclusion criteria were: (1) being complicated with severe pulmonary complications (such as active infection or pulmonary hemorrhage) that made exercise training unsuitable; (2) having other severe comorbidities (e.g., unstable cardiovascular disease, severe renal insufficiency) that might interfere with rehabilitation training or threaten patient safety; (3) having cognitive impairment leading to inability to understand instructions or cooperate with training; and (4) currently participating in other clinical trials of pulmonary rehabilitation or exercise interventions. No minimum interval between allo-HSCT and study enrollment was specified. Patients meeting the eligibility criteria were considered for enrollment irrespective of time since transplantation, provided that they were clinically stable and able to participate in the rehabilitation program. No formal *a priori* sample-size calculation was performed. All eligible patients meeting the study criteria during the predefined recruitment period were consecutively enrolled; therefore, the study was considered exploratory.

### Dumbbell-assisted diaphragmatic breathing training

Dumbbell-assisted diaphragmatic breathing training (DADBT) was performed using an adjustable-resistance breathing trainer [JT-WB20; JingTai Health Technology (Dongguan) Co., Ltd., Dongguan, Guangdong, China]. The device provides respiratory loading through an internal weight-based resistance mechanism. Participants inhaled slowly through the nose while allowing the abdominal wall to expand with diaphragmatic descent and subsequently exhaled slowly through the device mouthpiece against the selected resistance while actively contracting the abdominal wall. One complete inspiration–expiration sequence constituted one breathing cycle. Training was performed 3–4 times daily, with 8–10 breathing cycles per session, for 12 consecutive weeks. The training program was primarily performed at home after participants had received standardized instruction on the breathing technique and use of the device from the clinical team. The initial training procedure was demonstrated and checked by trained clinical staff to ensure that participants could perform the technique correctly. Subsequent daily training sessions were performed independently at home, with weekly clinical follow-up to reinforce correct technique and address training-related concerns.

### Outcome measures

The primary outcome was 6-minute walk distance (6MWD), selected as a practical measure of functional exercise capacity. The secondary outcome was bilateral handgrip strength, included as an indicator of peripheral muscle function in this post-transplant population. These outcomes were selected to assess clinically relevant functional consequences rather than the specific physiological effect of the intervention on the diaphragm. The 6-minute walk test (6MWT) was performed at baseline and after completion of the 12-week intervention. Participants were instructed to walk as far as possible during the 6-minute test period. Heart rate and peripheral oxygen saturation (SpO₂) were recorded immediately before and after the test, and the total distance walked was recorded in meters. Perceived dyspnea was assessed as an additional symptom measure immediately after the 6MWT using the modified Borg 0–10 scale, with higher scores indicating greater perceived breathlessness. Borg dyspnea scores were recorded at baseline and after completion of the 12-week intervention. No verbal encouragement was provided during testing. Bilateral handgrip strength was assessed using an electronic hand dynamometer (EH101; Zhongshan Camry Electronic Co., Ltd., Zhongshan, Guangdong, China). Participants stood upright with their arms relaxed alongside the body. Two maximal-effort trials were performed for each hand, and the highest value obtained for each hand was retained. The mean of the maximum values from the left and right hands was used as the participant-level handgrip strength measure and expressed in kilograms (kg). Training-related adverse events were monitored throughout the 12-week intervention and documented by the clinical team. Events of interest included symptoms or clinical events that required interruption, modification, or discontinuation of training.

### Exploratory analyses according to baseline characteristics

Exploratory analyses examined whether changes in the study outcomes were associated with selected baseline demographic and clinical characteristics. Age and the heart-rate response during the baseline 6MWT were analyzed as continuous variables. The heart-rate response was calculated as ΔHR = HRpost − HRpre (beats/min). Baseline ΔHR was included as an exploratory marker of the cardiovascular response to submaximal exercise, whereas supplemental oxygen requirement during the baseline 6MWT was included as a clinically relevant indicator that may reflect greater gas-exchange impairment or underlying disease severity. These variables were examined to explore whether baseline physiological or clinical status was associated with subsequent changes in 6MWD or handgrip strength; they were not intended as validated predictors of rehabilitation response. Sex and supplemental oxygen requirement were analyzed as categorical variables. These analyses were exploratory and were not prespecified as confirmatory analyses.

### Statistical analysis

Statistical analyses were performed using R software (version 4.2.2). Continuous variables were summarized as mean ± standard deviation (SD) when approximately normally distributed and as median with interquartile range (IQR) otherwise; categorical variables were summarized as number and percentage. The primary outcome was the within-participant change in 6MWD from baseline to 12 weeks (Δ6MWD), and the secondary outcome was the corresponding change in handgrip strength (ΔHGS). The distributions of paired change scores were assessed graphically and using the Shapiro–Wilk test. Approximately normally distributed paired differences were analyzed using paired *t*-tests; otherwise, Wilcoxon signed-rank tests were used. The change in post-6MWT Borg dyspnea score from baseline to week 12 was analyzed using the same distribution-based approach for paired observations. Effect estimates were reported with 95% confidence intervals (CIs).

Exploratory analyses assessed associations of Δ6MWD and ΔHGS with baseline age and baseline 6MWT heart-rate response (ΔHR) using Spearman's rank correlation coefficient (*ρ*). Differences in outcome changes according to sex and supplemental oxygen requirement were evaluated directly using Welch's two-sample *t*-test for approximately normally distributed change scores or the Mann–Whitney *U* test otherwise. Differential response between subgroups was inferred from direct between-group comparisons of change rather than from differences in the statistical significance of separate within-group tests. All statistical tests were two-sided, with *p* < 0.05 considered statistically significant.

## Results

### Baseline clinical characteristics

During the recruitment period, 58 patients with BOS after allo-HSCT were assessed for eligibility, of whom 27 were excluded before enrollment. Thirty-one patients were enrolled, completed the 12-week intervention and post-intervention assessment, and were included in the final analysis (Figure 1). Baseline demographic and clinical characteristics are summarized in Table 1. Nine participants (29.0%) required supplemental oxygen during the baseline 6MWT. Mean baseline 6MWD was 406.3 ± 117.9 m, mean bilateral handgrip strength was 23.2 ± 9.8 kg, and the median time from allo-HSCT to study enrollment was 39.0 months (IQR: 24.5–48.0 months).

**Figure 1:**
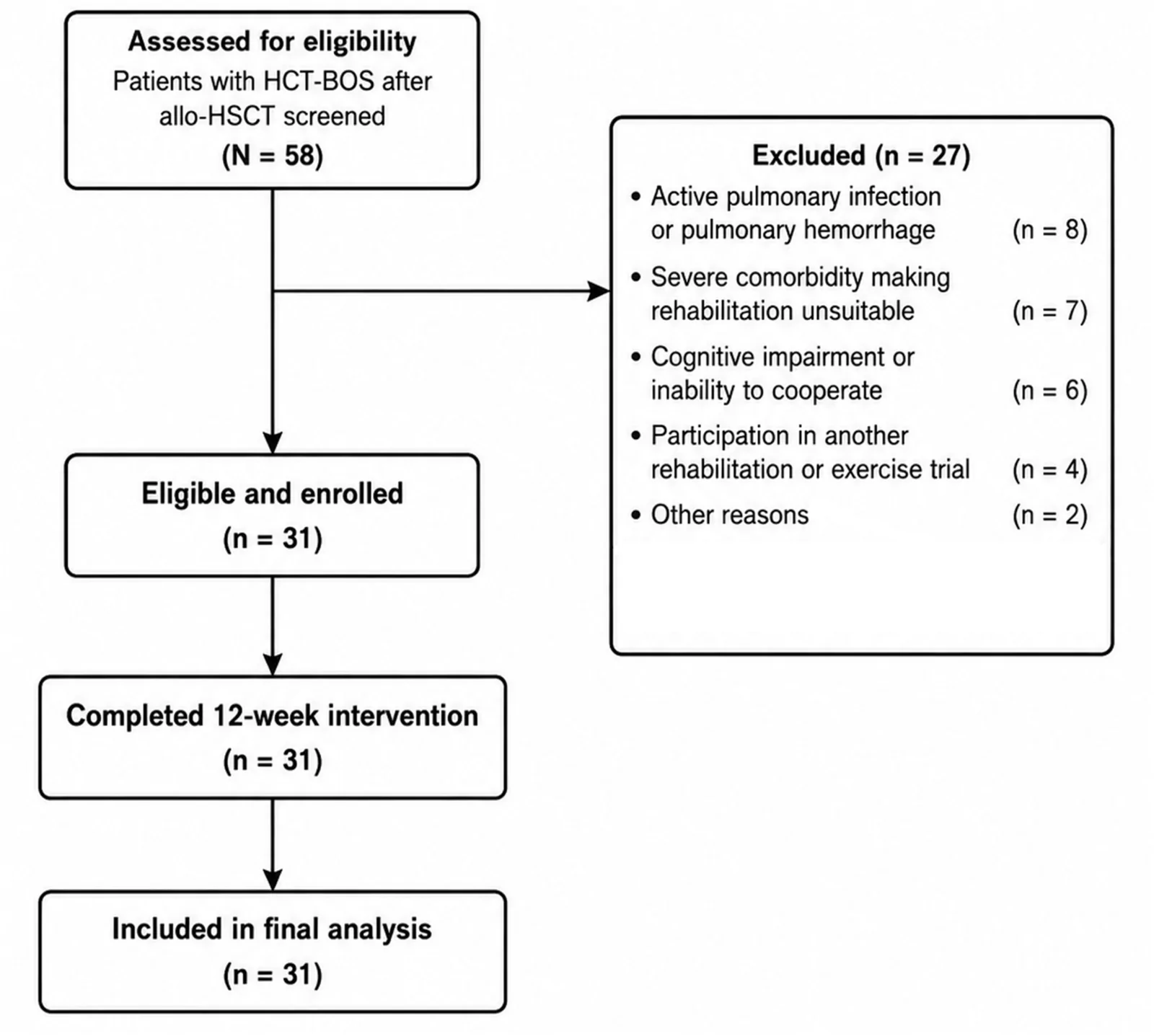
Flow diagram of participant screening, eligibility assessment, enrollment, and follow-up.

**Table 1:** Baseline demographic and clinical characteristics of the study participants.

Characteristics	Overall (*n* = 31)
Age, years	**36.8 ± 11.9**
Female sex, *n* (%)	17 (54.8)
Male sex, *n* (%)	14 (45.2)
BMI, kg/m^2^	19.6 ± 4.1
Time since allo-HSCT, months	39.0 (24.5–48.0)
Supplemental oxygen required during baseline 6MWT, *n* (%)	9 (29.0)
Supplemental oxygen not required during baseline 6MWT, *n* (%)	22 (71.0)
Baseline 6MWD, m	406.3 ± 117.9
Baseline bilateral handgrip strength, kg	23.2 ± 9.8

Values are mean ± standard deviation or *n* (%), except time since allo-HSCT, which is presented as median (interquartile range). BMI, body mass index; allo-HSCT, allogeneic hematopoietic stem cell transplantation; 6MWT, six-minute walk test; 6MWD, six-minute walk distance.

### Changes in functional outcomes following the intervention

Mean 6MWD increased from 406.3 m at baseline to 425.9 m at week 12, corresponding to a mean within-participant increase of 19.6 m (95% CI: 9.3–29.9 m; *p* < 0.001). Mean bilateral handgrip strength increased from 23.2 kg to 25.0 kg, corresponding to a mean increase of 1.8 kg (95% CI: 1.3–2.3 kg; *p* < 0.001). Mean post-6MWT Borg dyspnea score decreased from 4.1 ± 1.3 at baseline to 3.2 ± 1.2 at week 12, corresponding to a mean reduction of 0.9 points (95% CI: 0.5–1.3; *p* < 0.001).

The change in 6MWD did not differ significantly between women and men (12.3 ± 20.9 m vs. 28.5 ± 33.7 m; *p* = 0.282). In contrast, the increase in bilateral handgrip strength was greater in women than in men (2.3 ± 1.6 kg vs. 1.2 ± 0.7 kg; *p* = 0.021) (Figure 2).

**Figure 2:**
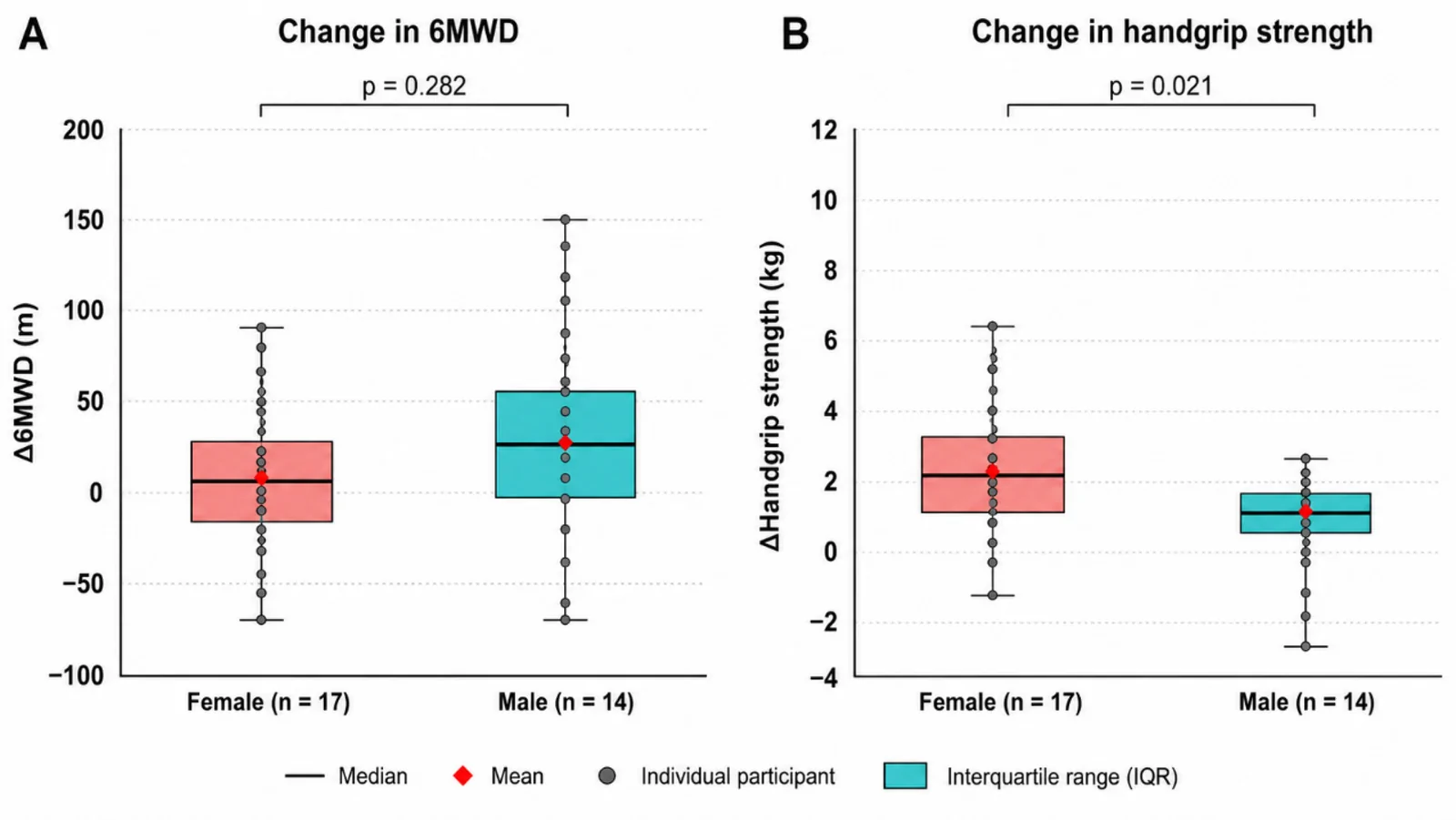
Changes in functional outcomes according to sex. **(A)** Change in 6MWD from baseline to week 12; **(B)** change in bilateral handgrip strength from baseline to week 12. Individual participants are shown as points. *P* values represent direct between-sex comparisons of change scores.

### Associations of baseline characteristics with outcome changes

Age was not significantly associated with Δ6MWD or ΔHGS (Figure 3). Baseline heart-rate response during the 6MWT was also not significantly associated with Δ6MWD or ΔHGS. Changes in 6MWD and handgrip strength did not differ significantly according to supplemental oxygen requirement during the baseline 6MWT (Table 2).

**Figure 3:**
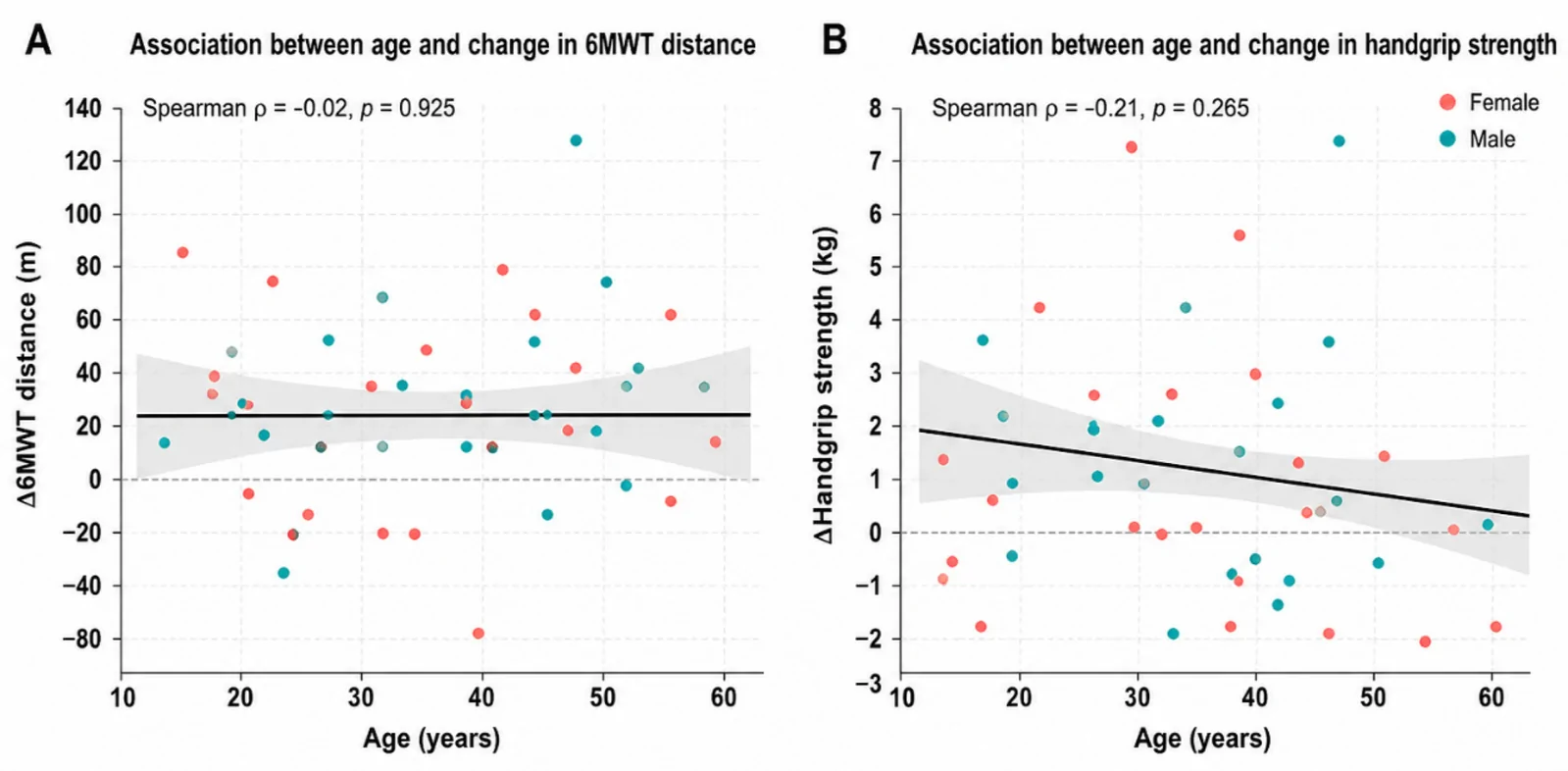
Associations between age and changes in functional outcomes. **(A)** Association between age and change in 6-minute walk distance (Δ6MWD); **(B)** association between age and change in bilateral handgrip strength (ΔHGS). Individual participants are shown as points and colored according to sex. Associations were assessed using Spearman's rank correlation coefficient (*ρ*).

**Table 2:** Exploratory associations of baseline heart-rate response and supplemental oxygen requirement with changes in functional outcomes.

Baseline characteristic	Group/analysis	*n*	Δ6MWD, m	Association/*p* value	ΔHGS, kg	Association/*p* value
Baseline 6MWT heart-rate response, ΔHR	Spearman correlation	31	—	*ρ* = −0.068, *p* = 0.717	—	*ρ* = −0.001, *p* = 0.996
Supplemental oxygen requirement during baseline 6MWT	No	22	21.1 ± 21.0		1.89 ± 1.51	
	Yes	9	15.9 ± 42.4	*p* = 0.156	1.54 ± 0.86	*p* = 0.794

Values for change scores are presented as mean ± SD for descriptive purposes. Δ6MWD, week-12 6MWD—baseline 6MWD; ΔHGS, week-12 handgrip strength—baseline handgrip strength. Associations between baseline ΔHR and outcome changes were assessed using Spearman's rank correlation coefficient. Differences in change scores between participants with and without supplemental oxygen requirement during the baseline 6MWT were assessed using the Mann–Whitney *U* test. 6MWT, 6-minute walk test; 6MWD, 6-minute walk distance; HGS, handgrip strength; ΔHR, difference between heart rate immediately after and before the baseline 6MWT.

### Safety analysis

No serious training-related adverse events were observed during the 12-week intervention. Two participants experienced mild fatigue during the initial phase of training; symptoms resolved after 10–15 min of rest and did not prevent subsequent participation. No training-related falls, muscle strains, arrhythmias, pulmonary infections, or episodes of aggravated chest tightness or dyspnea were recorded.

## Discussion

In this prospective single-arm study of patients with BOS after allo-HSCT, 12 weeks of dumbbell-assisted diaphragmatic breathing training was followed by improvements in functional exercise capacity, handgrip strength, and perceived exertional dyspnea. No serious training-related adverse events were observed. Although the absence of a concurrent control group precludes causal attribution, the direction of these findings is consistent with previous evidence suggesting that rehabilitation may improve functional status after allo-HSCT and in patients with BOS (12, 25).

The observed increase in 6MWD was consistent with improved functional exercise capacity over the 12-week study period. Tran et al. (12) reported improvement in 6MWD following pulmonary rehabilitation in patients with BOS after HSCT, while Barğı et al. (25) demonstrated improved 6MWD and respiratory-muscle strength following inspiratory-muscle training in allo-HSCT recipients. These interventions differed from the present program in modality, intensity, and duration, and direct quantitative comparison is therefore limited. Nevertheless, the findings collectively support the potential role of structured rehabilitation in addressing exercise intolerance after transplantation. In the present cohort, the mean increase in 6MWD was 19.6 m. This change was statistically significant but smaller than the approximately 30-m minimal important difference proposed for adults with chronic respiratory disease (28). Because no BOS-specific threshold has been established after allo-HSCT and the present cohort included both adolescents and adults, the clinical importance of the observed improvement remains uncertain.

The observed increase in handgrip strength is also noteworthy because the intervention was directed primarily at breathing mechanics rather than peripheral resistance training. Exercise limitation after allo-HSCT is likely multifactorial, with previous studies implicating both impaired skeletal-muscle oxygenation and relationships between muscle oxygen extraction, pulmonary function, and exercise tolerance (29, 30). Handgrip strength is a practical indicator of peripheral physical function and may be influenced by physical activity and deconditioning, nutritional status, disease-related factors, and treatment-related changes in muscle function after allo-HSCT (31–33). One possible explanation is that improved breathing efficiency or reduced exertional dyspnea may have facilitated greater daily physical activity during the intervention period, indirectly contributing to peripheral functional improvement. However, this mechanism was not directly assessed.

The reduction in post-exercise Borg dyspnea scores provides additional symptom-level context for the improvement in walking performance. Reductions in exertional dyspnea during pulmonary rehabilitation may occur alongside improved exercise tolerance, although the underlying mechanisms can include changes in conditioning, breathing pattern, respiratory sensation, and other physiological or perceptual factors (20, 34). However, direct physiological measurements such as maximal inspiratory pressure, diaphragm excursion, diaphragm thickness, respiratory-muscle endurance, or cardiopulmonary exercise testing were not performed. Accordingly, the present study cannot determine whether the functional changes were mediated by improved diaphragmatic function, altered ventilatory mechanics, reduced dynamic hyperinflation, cardiovascular adaptation, peripheral-muscle factors, or a combination of these mechanisms.

The exploratory analyses of baseline characteristics were hypothesis-generating and should be interpreted in the context of the small sample size. No consistent associations were identified between functional changes and age, baseline heart-rate response, or supplemental oxygen requirement. These exploratory findings do not establish the absence of clinically meaningful effect modification and should primarily inform the design of larger studies rather than be considered definitive subgroup findings.

A practical feature of the intervention is its portability and suitability for repeated home-based practice. Home-based pulmonary rehabilitation may reduce barriers associated with repeated attendance at rehabilitation facilities (23). However, adherence was not objectively quantified in the present study; therefore, the practical feasibility of home-based delivery should not be interpreted as evidence of superior adherence or effectiveness compared with conventional center-based pulmonary rehabilitation.

### Limitations

Several limitations should be considered. First, this was a single-center, single-arm study without a concurrent control group; therefore, the observed pre–post changes cannot be attributed exclusively to dumbbell-assisted diaphragmatic breathing training. Natural variation in BOS, concurrent medical treatment, behavioral changes, recovery over time, and repeated testing may have contributed to the findings. Second, the sample size was small, particularly for exploratory analyses of potential effect modifiers, resulting in imprecise estimates and limited statistical power. In addition, extrapulmonary manifestations of cGVHD that may affect muscle function, such as joint contractures or fascial involvement, and treatment-related complications such as glucocorticoid-associated myopathy were not systematically assessed. These unmeasured factors may have influenced baseline handgrip strength or its change over time and therefore represent potential confounders of the observed handgrip-strength findings. Third, the 6MWT procedure did not use standardized verbal encouragement, and duplicate tests to account for the recognized learning effect were not documented; 6MWD is sensitive to these methodological factors. Fourth, the study included both adolescents and adults, limiting direct application of adult-specific reference values and clinically important difference thresholds to the entire cohort. Fifth, respiratory muscle strength, diaphragm function, and longitudinal pulmonary-function changes were not directly assessed, limiting mechanistic interpretation. Sixth, adherence to the home-based training program was not objectively quantified. Finally, long-term follow-up was not performed, so the durability of the observed changes remains unknown. Larger randomized controlled trials incorporating standardized exercise testing, pulmonary-function assessment, cardiopulmonary exercise testing, respiratory-muscle measurements, objective adherence monitoring, and longer-term follow-up are warranted to establish efficacy and better characterize the physiological determinants of exercise limitation and response to rehabilitation.

## Conclusion

In this prospective single-arm study of 31 patients with BOS after allo-HSCT, 12 weeks of dumbbell-assisted diaphragmatic breathing training was associated with improvements in functional exercise capacity and handgrip strength, with no serious training-related adverse events observed. However, the absence of a concurrent control group, the small sample size, limitations in outcome-measure standardization, and the exploratory nature of analyses of baseline characteristics preclude definitive conclusions regarding treatment efficacy or differential response among patient subgroups. The clinical importance of the observed changes also remains uncertain. Larger randomized controlled trials incorporating standardized 6MWT procedures, pulmonary-function assessment, respiratory-muscle measurements, objective adherence monitoring, and longer-term follow-up are warranted.

## Data Availability

The original contributions presented in the study are included in the article/Supplementary Material, further inquiries can be directed to the corresponding author.
